# HILI Inhibits TGF-β Signaling by Interacting with Hsp90 and Promoting TβR Degradation

**DOI:** 10.1371/journal.pone.0041973

**Published:** 2012-07-27

**Authors:** Kun Zhang, Yilu Lu, Ping Yang, Chao Li, Huaqin Sun, Dachang Tao, Yunqiang Liu, Sizhong Zhang, Yongxin Ma

**Affiliations:** 1 Department of Medical Genetics, State Key Laboratory of Biotherapy, West China Hospital, Sichuan University, Chengdu, China; 2 Sichuan University-The Chinese University of Hong Kong Joint Laboratory for Reproductive Medicine, West China Institute of Women and Children’s Health, West China Second University Hospital, Sichuan University Chengdu, China; University of Hong Kong, Hong Kong

## Abstract

PIWIL2, called HILI in humans, is a member of the PIWI subfamily. This subfamily has highly conserved PAZ and Piwi domains and is implicated in several critical functions, including embryonic development, stem-cell self-renewal, RNA silencing, and translational control. However, the underlying molecular mechanism remains largely unknown. Transforming growth factor-β (TGF-β) is a secreted multifunctional protein that controls several developmental processes and the pathogenesis of many diseases. TGF-β signaling is activated by phosphorylation of transmembrane serine/threonine kinase receptors, TGF-β type II (TβRII), and type I (TβRI), which are stabilized by Hsp90 via specific interactions with this molecular chaperone. Here, we present evidence that HILI suppresses TGF-β signaling by physically associating with Hsp90 in human embryonic kidney cells (HEK-293). Our research shows that HILI mediates the loss of TGF-β-induced Smad2/3 phosphorylation. We also demonstrate that HILI interacts with Hsp90 to prevent formation of Hsp90-TβR heteromeric complexes, and improves ubiquitination and degradation of TβRs dependent on the ubiquitin E3 ligase Smurf2. This work reveals a critical negative regulation level of TGF-β signaling mediated by HILI (human PIWIL2) by its ability to interact with Hsp90 and promote TβR degradation.

## Introduction

The PIWI proteins are found in a wide variety of animals, from Drosophila to humans. They include two well-conserved domains (Piwi and PAZ domains) [1]–[3]. These proteins are highly conserved during evolution and play pivotal roles in stem-cell self-renewal, cell cycling, gametogenesis, RNA silencing, epigenetic modulation, chromatin remodeling, and translation control in diverse organisms [2], [4]–[11]. In Drosophila, genetic studies have shown that *piwi* is required for germline development, downstream gametogenic functions, and canalization [11]–[14]. The *piwi* genes in zebrafish (*ziwi, zili*) control transposon defense, early embryogenesis, and the development of the embryonic genital ridge and gonads [10], [15], [16]. In mice, mutations of *piwi* genes (*miwi*, *mili*, and *miwi2*) influence the meiotic progression of developing sperm, finally resulting in dyszoospermia [17]–[19]. In human, four members of the PIWI subfamily, *piwil1/hiwi, piwil2/hili, piwil3*, and *piwil4/hiwi2*, have been identified. They are located on 12q23, 8p24, 22q11.2, and 11q12, respectively [3]. All members of the PIWI subfamily are mainly expressed in the testis or embryo [3], [7], [12], [16]. Recent studies show that *hili* (human *piwil2* gene) functions in a diverse set of cellular processes and may be involved in signaling regulation, but the underlying molecular mechanisms are largely unknown [20]–[23].

The transforming growth factor-β (TGF-β) signaling pathway contributes to the regulation of early development, the cell cycle, differentiation, hematopoesis, angiogenesis, chemotaxis, immune functions, and tumorigenesis [24]–[27]. TGF-β exerts its function by inducing phosphorylation of receptor-activated Smad [28]–[30]. The TGF-β signaling is primarily transduced by a pair of transmembrane serine/threonine kinase receptors, the TGF-β type II receptor (TRβII), and type I receptor (TβRI). TβRII is constitutively active and phosphorylates the TβRI in response to TGF-β signaling [31], [32]. R-Smad is then phosphorylated by the activated TβRI. Phosphorylated R-Smad forms a complex with Smad4, which is then translocated into the nucleus, where it binds specific Smad-binding element (SBE) to cause ligand-induced changes in the transcription of a variety of genes in a context-dependent manner [33]–[35].

Hsp90, 90-kDa heat-shock protein, regulates a wide variety of signaling pathways [36]–[41]. Recent studies have shown that Hsp90 can stabilize TGF-β receptors and prevent ubiquitin-mediated degradation of TβRs [42]–[44]. Here, we first reveal that HILI is a novel negative regulator of the TGF-β signaling pathway by competing with TβRs for Hsp90 and promoting TβR degradation. HILI binds Hsp90 to prevent formation of Hsp90-TβR complexes, improves degradation of TβRs dependent on the ubiquitin E3 ligase Smurf2, and finally blocks Smad2/3 phosphorylation to inhibit TGF-β signaling. Considering that Hsp90 is also involved in a wide variety of other signaling pathways, this work provides new perspective on the study of the participation of PIWI proteins in regulating diverse types of signal transductions, extending the function of the PIWI subfamily.

## Results

### HILI Abrogates TGF-β Signaling at the Level of Smad Phosphorylation and Inhibits Cells Apoptosis

Smad proteins are key transducers in TGF-β signaling, and TGF-β induces Smad2/3 phosphorylation to regulate a cascade of downstream events [45]. Cyclin-dependent kinase inhibitor p21, which is regulated by phosphorylated Smad2/3, has an effect on TGF-β-induced growth arrest [46]. Expression of plasminogen activator inhibitor-1 PAI-1 and Smad7 are also induced by TGF-β signaling [47]–[49]. To establish whether HILI controls TGF-β signaling, we used several well-established approaches to investigate the effects of HILI on TGF-β-induced expression of p21, PAI-1 and Smad7, and Smad2/3 phosphorylation.

**Figure 1:**
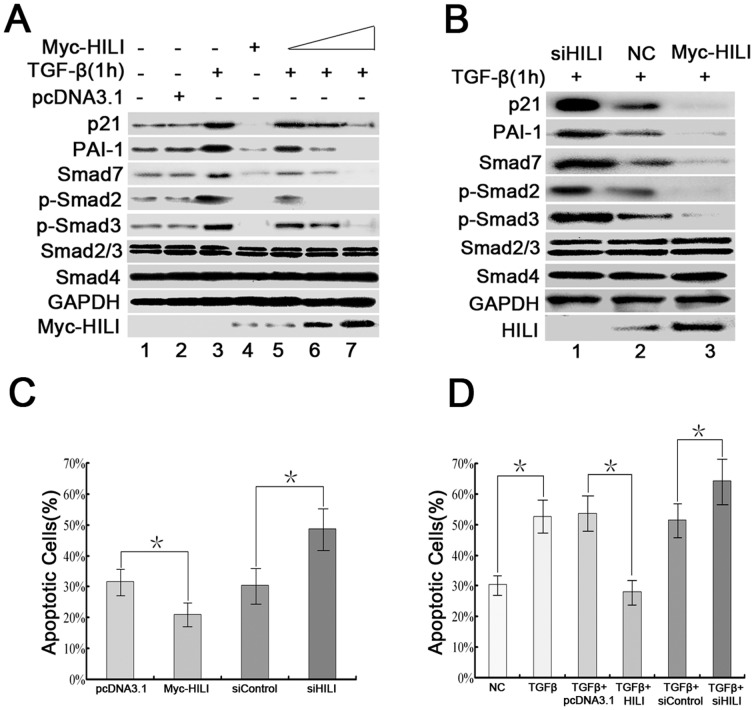
Effects of HILI on TGF-β signaling at the level of Smad phosphorylation and ultimate effect on apoptosis. ***A*** **.** HILI reduces expression of, PAI-1, and Smad7 and prevents Smad2/3 activation. HEK-293 cells were transfected with Myc-HILI at a concentration gradient (0.5 µg, 0.5 µg, 1.5 µg, and 3 µg per well) and treated with TGF-β for 1 h before cells were harvested as indicated. Cells lysates were used for Western blot analysis with anti-, anti-PAI-1, anti-phospho-Smad2, anti-phospho-Smad3, anti-Smad2/3, anti-Smad4, anti-Smad7, anti-GAPDH, and anti-Myc antibodies. ***B*** **.** siHILI reverses the reduction of p21, PAI-1, Smad7, and phosphorylated Smad2/3 mediated by HILI. HEK-293 cells were transfected with siHILI or Myc-HILI, after 48 h, treated with TGF-β for 1 h before harvesting. They were then used in Western blot analysis by using antibodies as in (*A*). NC represents negative control transfected with siControl. ***C*** **and** ***D*** **.** HILI inhibits TGF-β-induced cell apoptosis. HEK-293 cells were respectively transfected with pcDNA3.1, Myc-HILI, siControl and siHILI, and treated with TGF-β for 12 h before cells were harvested as indicated. After transfection 48 h, apoptosis of cells was analyzed with Annexin V/PI double staining and flow cytometry. NC represents negative control without any treatments. Each experiment was performed in triplicate. *indicates *P<*0.05.

Initially, we transfected expression vector encoding HILI protein into HEK-293 cells on a concentration gradient. Western blot analysis showed that the expression of p21, PAI-1, and Smad7 and the phosphorylation of Smad2/3 were inhibited or even abolished by HILI in a dose-dependent manner. This occurred despite TGF-β stimulation (Fig. 1*A*, lanes 4–7).

To confirm that the loss of p21, PAI-1, Smad7, and phosphorylated Smad2/3 proteins is due to HILI, we reduced endogenous levels of HILI by using siRNA (siHILI) and assessed the effect on the expression of p21, PAI-1, and Smad7 and on the activation of Smad2/3 induced by TGF-β. siHILI increased the expression of p21, PAI-1, and Smad7, and Smad2/3 phosphorylation (Fig. 1*B*, lanes 1 and 2). We then increased the expression level of HILI by transfecting vector expressing HILI. This ectopic expression of HILI decreased levels of p21, PAI-1, Smad7, and phosphorylated Smad2/3 (Fig. 1*B*, lanes 2 and 3). Above results suggest that HILI abrogates TGF-β signaling at the level of Smad phosphorylation.

We then checked effects of HILI on apoptosis. Fluorescence-activated cell sorter (FACS) analysis showed a significant decrease in apoptosis, from 31.7% to 21.1%, in HEK-293 cells overexpressing HILI. Apoptosis increased to 48.7% in HILI-knockdown cells (Fig. 1*C*). Notably, TGF-β-induced apoptosis obviously decreased, from 53.7% to 28.1%, in the cells overexpressing HILI. TGF-β-induced apoptosis increased to 64.3% in HILI-knockdown cells (Fig. 1*D*). These findings indicate that HILI inhibits TGF-β-induced apoptosis. Coupled with our data showing that HILI abrogates TGF-β signaling, these findings suggest that HILI inhibits cell apoptosis by abrogating TGF-β signaling.

### HILI Promotes Degradation of TβRs

**Figure 2:**
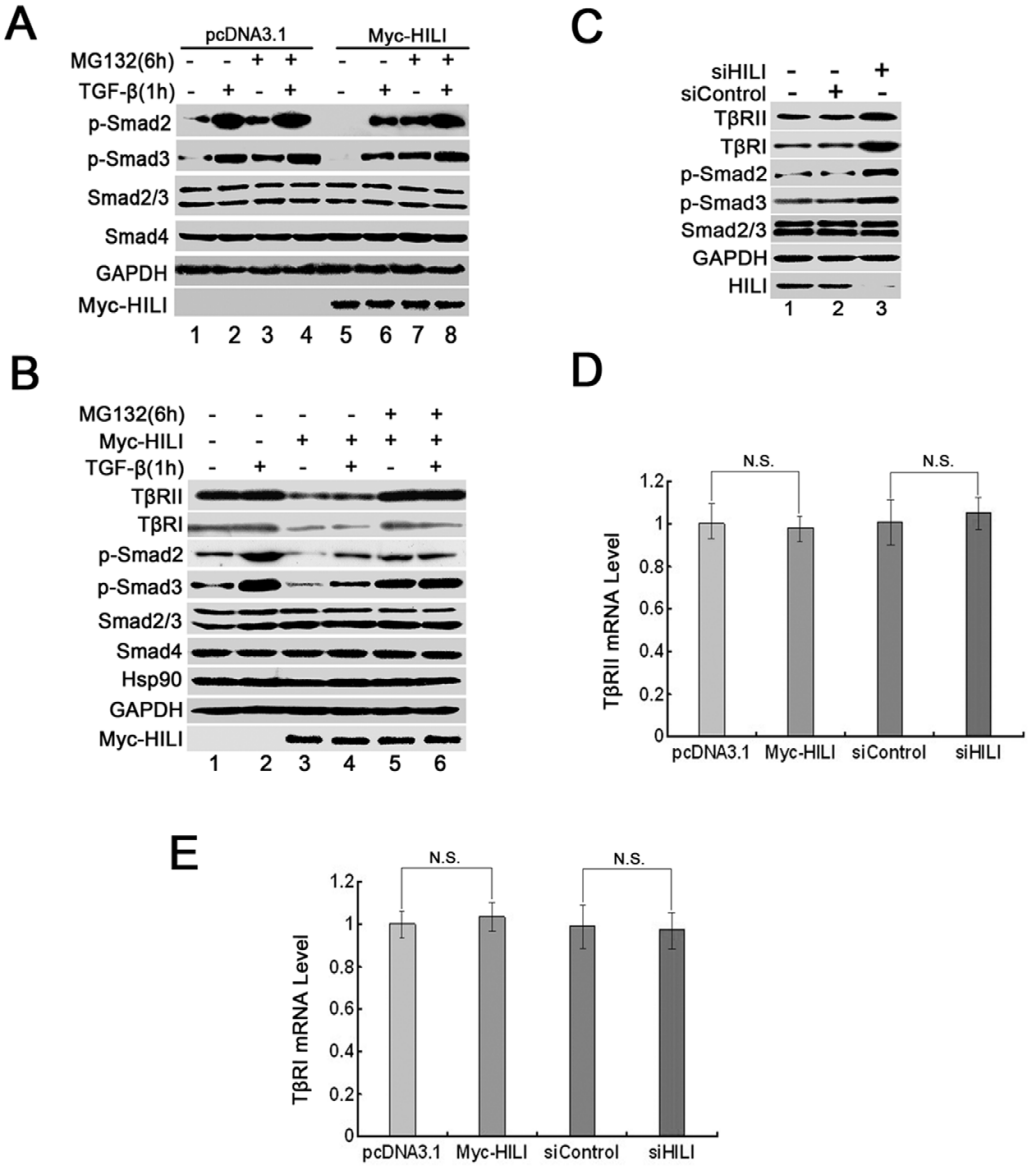
Effects of HILI on degradation of TβRs and Smad. *A*. HILI-mediated loss of TGF-β-induced Smad activation is restored by MG132. HEK-293 cells were transfected with Myc-HILI or pcDNA3.1. After 48 h, they were treated with MG132 for 6 h. TGF-β was added for the last 1 h, as indicated. Cells were harvested for Western blotting using anti-phospho-Smad2, anti-phospho-Smad3, anti-Smad2/3, anti-Smad4, anti-GAPDH, and anti-Myc antibodies. ***B*** **.** HILI promotes TβR degradation. HEK-293 cells were transfected with Myc-HILI. After 48 h, the cells were treated as in (*A*). Cell lysates were used for Western blotting with anti-TβRII, anti-TβRI, anti-phospho-Smad2, anti-phospho-Smad3, anti-Smad2/3, anti-Smad4, anti-Hsp90, anti-GAPDH, and anti-Myc antibodies. ***C*** **.** siHILI improves TβR protein levels. HEK-293 cells were transfected with siControl or siHILI. After 48 h, cell lysates were used for Western blotting with anti-TβRII, anti-TβRI, anti-phospho-Smad2, anti-phospho-Smad3, anti-Smad2/3, anti-GAPDH, and anti-HILI antibodies. ***D*** and ***E.*** Neither HILI nor siHILI was found to affect TβR mRNA levels. HEK-293 cells were transfected with pcDNA3.1, Myc-HILI, siControl, and siHILI as indicated. After 48 h, the cells were harvested for total RNA extraction and then for quantitative RT-PCR. Each experiment was performed in triplicate. N.S. indicates *P>*0.05.

Results have shown that HILI suppresses Smad2/3 phosphorylation to block TGF-β signaling and decreases cell apoptosis. To explore the molecular mechanism underlying HILI-mediated loss of TGF-β-induced Smad2/3 phosphorylation, we determined whether HILI could promote the degradation of Smad or TGF-β receptors and ultimately inhibit Smad2/3 phosphorylation. HEK-293 cells were transfected with vectors expressing HILI proteins. The cells were treated with proteasome inhibitor MG132 before TGF-β stimulation as shown in Fig.2. HILI overexpression did not change the level of Smad2/3/4 expression. However, Smad2/3 phosphorylation was noticeably weakened by HILI independently of TGF-β stimulation (Fig. 2*A*, lanes 1, 2, 5, and 6). The loss of Smad2/3 phosphorylation regulated by HILI was rescued by MG132 treatment (Fig. 2*A*, lanes 7 and 8). These results suggest that HILI blocks Smad2/3 activation in a proteasome-dependent manner. Degradation was not found to occur at the level of Smad but rather upstream of phosphorylated Smad2/3.

We reasoned that the degradation event must be located upstream of phosphorylated Smad2/3, so we measured the effect of HILI on TβR stability. HEK-293 cells were transfected with expression vectors encoding HILI. Some of these cells were treated with the proteasome inhibitor MG132 before TGF-β stimulation. In transfected cells, we observed that both TβRII and TβRI protein levels were profoundly reduced by HILI, paralleling HILI-mediated loss of phosphorylated Smad2/3, although Smad2/3/4 protein levels remained stable (Fig. 2*B*, lanes 3 and 4). Significantly, the reductions of TβRs and phosphorylated Smad2/3 regulated by HILI were recovered by MG132 treatment (Fig. 2*B*, lanes 5 and 6). We then knocked down HILI and observed that the TβR protein levels were increased by siHILI (Fig. 2*C*). However, quantitative RT-PCR results showed that HILI did not affect TβR mRNA levels (Figs. 2*D and 2E*). These findings suggest that HILI weakens the stability of TβR proteins, promotes degradation in a proteasome-dependent manner, and blocks Smad2/3 phosphorylation, which results in the inhibition of TGF-β signaling.

### HILI Interacts with Hsp90 to Block Formation of Hsp90-TβR Complexes

**Figure 3:**
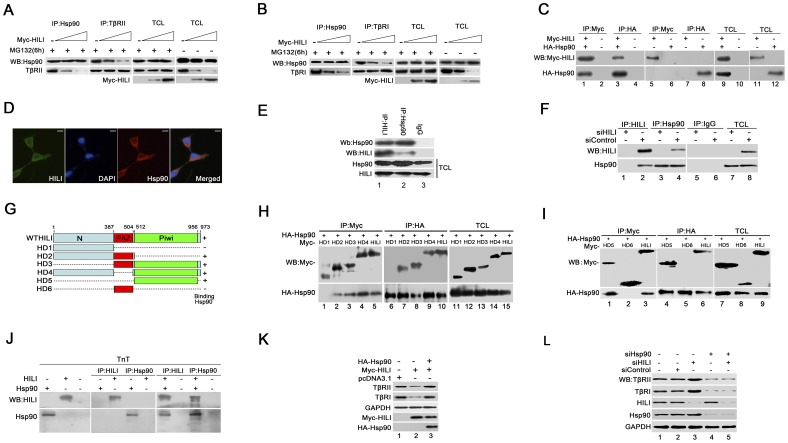
Effects of interaction between HILI and Hsp90 on the formation of Hsp90-TβR complexes. ***A*** and ***B*** **.** HILI impairs interaction between Hsp90 and TβRs. HEK-293 cells were transfected with Myc-HILI at a concentration gradient (1.5 µg and 3 µg per well). After 48 h of culture, cells were treated with MG132 for 6 h where indicated. The lysates were subjected to anti-Hsp90 or anti-TβRs (TβRII, TβRI) IP. Interactions between Hsp90 and TβRs were detected by (*A*) anti-Hsp90 and anti-TβRII or (*B*) anti-TβRI immunoblotting. Myc-HILI expression was detected using anti-Myc. TCL indicates total-cell lysates. ***C*** **–** ***I*** **.** HILI interacts with Hsp90 *in vivo*. ***C*** **.** Exogenous Myc-HILI binds HA-Hsp90. HEK-293 cells were cotransfected with Myc-HILI and HA-Hsp90. After 48 h of cell culture, Myc-HILI and HA-Hsp90 were coimmunoprecipitated (Co-IP) by anti-Myc or anti-HA. Then Western blotting was using to evaluate any interactions. ***D*** **.** Both HILI and Hsp90 are localized in cytoplasm. HEK-293 cells were transfected as in (*C*). The cells were harvested for immunofluorescence with anti-Myc and anti-HA antibodies. *Scale bars*, 10 µm. ***E*** **.** Endogenous interactions between HILI and Hsp90 in HEK-293 cells. Co-IP was performed with anti-HILI or anti-Hsp90, followed by Western blotting. ***F.*** The bands of Hsp90 and HILI are specific. HEK-293 cells were transfected with siControl or siHILI. The cell lysates were used for Co-IP by anti-HILI and anti-Hsp90. ***G*** **.** Different HILI deletion mutants. We constructed various HILI mutants by segmented-PCR and fusion PCR. All mutants were cloned into expression vector pcDNA3.1+Myc. ***H*** and ***I*** **.** Interaction between different HILI mutants and Hsp90. HEK-293 cells were cotransfected with Myc-HILI deletion mutants and HA-Hsp90. Co-IP as in (*C*). ***J.*** HILI interacts directly with Hsp90 *in vitro*. *In vitro* cell-free HILI and Hsp90 protein expression were carried out in two reactions using TnT® system, separately. The HILI and Hsp90 TnT® reactions were mixed together in binding buffer with protease inhibitors and incubated on a rotating platform at 4°C for 3h. Interaction between single HILI and Hsp90 protein was detected by Co-IP and Western blotting using anti-HILI and anti-Hsp90. ***K*** **.** Reduction of TβRs mediated by HILI was rescued by overexpression of Hsp90. Cell lysates were used for Western blotting with anti-TβRII, anti-TβRI, anti-GAPDH, anti-Myc, and anti-HA. ***L.*** siHILI increases TβR levels, but siHsp90 reduces them. HEK-293 cells were transfected with siControl, siHILI, and siHsp90 as indicated. After 48 h, cell lysates were used for Western blotting with anti-TβRII, anti-TβRI, anti-GAPDH, anti-HILI, and anti-Hsp90.

We began to investigate how HILI regulates TβR degradation. In the TGF-β signaling pathway, Hsp90 physically forms a complex with TβR to stabilize TβRII and TβRI [42]. We asked whether HILI could dismiss the endogenous interaction between Hsp90 and TβR to weaken TβR stability. Immunoprecipitation experiments showed that the amount of TβR coimmunoprecipitated with Hsp90 gradually dropped as the amount of HILI in HEK293 cells increased, although, in total lysates, neither TβR nor Hsp90 levels were different from those of MG132-treated cells (Figs. 3*A and 3B*).

HILI blocked the formation of Hsp90-TβR complexes, so we reasoned that HILI might reduce Hsp90 levels or compete with TβRs for this molecular chaperone, preventing the complexes from forming. To test this hypothesis, we first assessed whether HILI could affect the expression level of Hsp90. As shown in Fig. 2*B*, Hsp90 levels were not affected by ectopic expression of HILI and remained at the steady state. Next, we examined whether HILI could interact with Hsp90. Coimmunoprecipitation experiments revealed that HILI bound to Hsp90 (Fig. 3*C*, lanes 1 and 3) in HEK-293 cells transfected with Myc-HILI and HA-Hsp90. Immunofluorescence experiments showed that HILI was located mostly in the cytoplasm and overlapped with Hsp90 (Fig. 3*D*). To confirm the physical interaction between HILI and Hsp90, we carried out reciprocal immunoprecipitation of endogenous HILI and Hsp90 in HEK-293 cells. Immunoprecipitation and Western blot analyses revealed that HILI coprecipitated with Hsp90 (Fig. 3*E*), but not with TβRs (Fig. S1 and S2). The bands of Hsp90 and HILI were specific (Fig. 3*F*), we knocked down the expression of HILI and found that Hsp90 was not coimmunoprecipitated (Fig. 3*F*). These data show that HILI can interact with Hsp90. To further demonstrate interaction between HILI and Hsp90, we constructed several HILI protein deletion mutants (Fig. 3*G*). HEK-293 cells were transfected with Myc-HILI mutants and HA-Hsp90. Coimmunoprecipitation experiments showed that the HILI mutants HD1 (N-terminus) and HD6 (PAZ domain) failed to bind Hsp90, but other mutants, including HD2 (N-terminus and PAZ domain), HD3 (PAZ and Piwi domain), HD4 (N-terminus and Piwi domain), and HD5 (Piwi domain) remained able to bind Hsp90 (Figs. 3*H and 3I*). Single N-terminus or PAZ domain failed to bind Hsp90, while HD2 (N-terminus and PAZ domain) and other mutants including Piwi domain can interact with Hsp90. Hsp90 is a chaperone, and interaction mechanisms between proteins and Hsp90 are very complicated. Here, we focus whether HILI interact with Hsp90. Above data support that HILI can interact with Hsp90.

Furthermore, we asked whether HILI could interact directly with Hsp90, therefore proteins *in vitro* binding assay was used to detect direct interaction between that HILI and Hsp90 protein by TnT® Quick Coupled *in vitro* transcription/translation system (Promega) according to the manufacturers instructions. Our result showed that single HILI and Hsp90 protein co-immunoprecipitated *in vitro* (Fig.3*J*). This suggests that HILI can interact directly with Hsp90 *in vitro*.

To confirm our hypothesis, we determined whether Hsp90 could abolish the effect of HILI on TβRs. Western blotting showed that overexpression of Hsp90 rescued the TβR reduction mediated by HILI, as expected (Fig. 3*K*). Knockdown of both HILI and Hsp90 was also examined. We transfected siHILI and siHsp90 into HEK-293 cells. TβR levels were increased in HILI-knockdown cells, but the levels were reduced in all Hsp90-knockdown cells (Fig. 3*L* lanes 3–5).

These data indicate that Hsp90 is critical for participation of HILI in regulating TGF-β signaling. HILI interacts with Hsp90 rather than altering its expression level. HILI blocks formation of Hsp90-TβR complexes via interacting with Hsp90 and competing with TβRs for Hsp90 to impair TβR stability and promote the degradation, and finally negatively regulates TGF-β signaling.

### Smurf2 Is Required for HILI-Mediated TβR Degradation

**Figure 4:**
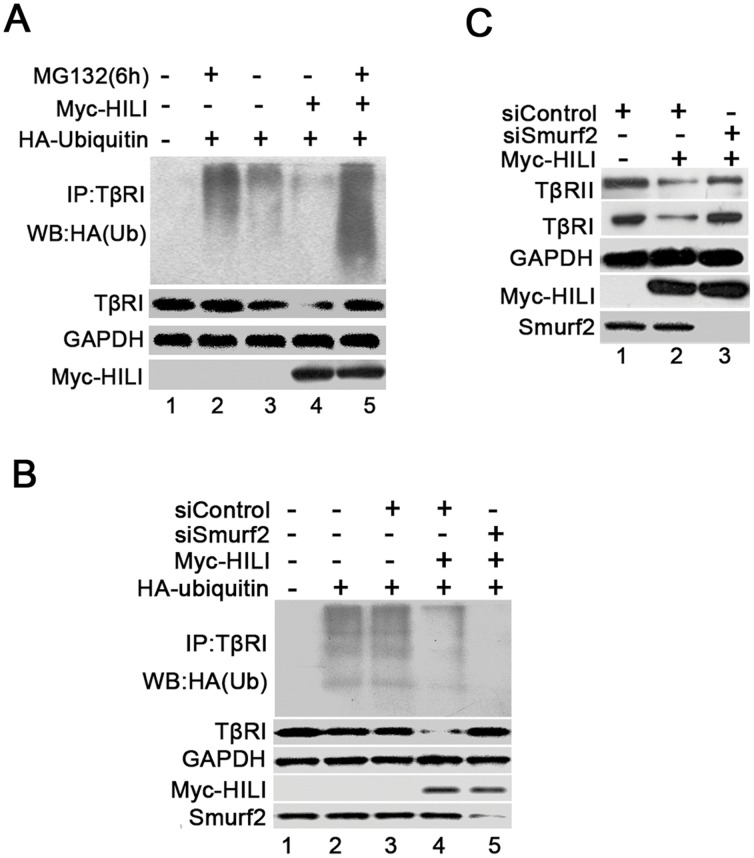
Role of smurf2 is required for HILI-regulated TβR degradation. ***A***. HILI improves TβRI ubiquitination. HEK-293 cells were cotransfected with Myc-HILI and HA-Ubiquitin, after 48 h, treated with MG132 for 6 h, TGF-β for the last 1 h as indicated. TβRI was precipitated with anti-TβRI antibody, then ubiquitination and degradation of TβRI were determined by anti-HA (Ub) and anti-TβRI immunoblotting. ***B.*** Smurf2 knockdown abolishes HILI-regulated TβRI ubiquitination. HEK-293 cells were cotransfected with Myc-HILI, HA-Ubiquitin, siSmurf2, and siControl where indicated. TβRI ubiquitination was checked as in (*A*). ***C*** **.** Smurf2 knockdown blocks HILI-regulated TβR degradation. HEK-293 cells were cotransfected with Myc-HILI, siSmurf2 and siControl where indicated. After 48 h, the lysates were used for Western blotting by anti-TRβII, anti-TβRI, anti-GAPDH, anti-Smurf2, and anti-Myc.

TβRs are known as the targets of ubiquitin-controlled degradation [43], [44]. Therefore we first determined whether HILI could enhance TβR ubiquitination. Endogenous TβRI was immunoprecipitated from HEK-293 cells transfected with Myc-HILI and HA-ubiquitin using TβRI antibody, and ubiquitination of TβRI was detected using anti-HA(Ub) Western blotting. Transfection with HILI alone promoted dramatic degradation of TβRI, so less ubiquitination was detected compared with controls (Fig. 4*A*, lanes 3 and 4). The addition of MG132 nearly recovered TβRI to original levels in the presence of HILI and facilitated the detection of TβRI ubiquitination. Notably, ectopic expression of HILI promoted TβRI ubiquitination (Fig. 4*A*, lanes 2 and 5). These results imply that HILI increases TβRI ubiquitination, and then improves its degradation controlled by ubiquitin.

Smurf2 is an E3 ubiquitin ligase responsible for degradation of both TRβII and TβRI [43], [44]. For this reason, we determined whether Smurf2 is necessary to HILI-mediated ubiquitination and degradation of TβRs. Endogenous expression of Smurf2 was inhibited by transfecting effective siSmurf2 into HEK-293 cells. siSmurf2 abolished HILI-mediated ubiquitination of TβRI (Fig. 4*B* *,* lanes 4 and 5). We did not observe HILI-mediated degradation of either TRβII or TβRI in Smurf2 knockdown cells (Fig. 4*C*). These findings suggest that Smurf2 is essential to HILI-mediated TβR degradation.

## Discussion

To the best of our knowledge, this is the first study to show that human PIWIL2 (HILI) inhibits TGF-β signaling by interacting with Hsp90 and enhancing the ubiquitin-controlled degradation of TβRs. As described by Sun *et al*., PIWIL2 in zebrafish (ZILI) interacts with Smad4 to prevent the formation of Smad2/3/4 complexes, and inhibits TGF-β signaling [10]. The said study on zebrafish PIWIL2 (ZILI), inspired us to determine whether human PIWIL2 (HILI) could physically associate with Smad4. Unexpectedly, the results of interaction assays showed that HILI was not a binding partner of Smad2/3/4 (Fig. S3). As shown by Sun *et al.*, the first 50 amino acids at N-terminus of the ZILI protein make up a functionally important region for binding to Smad4 [10]. HILI lacks these 50 amino acids, as indicated by homology analysis (data not shown). This may explain why HILI, the human homolog protein of ZILI, cannot interact with Smad4. The present study shows that HILI engages in regulating TGF-β signaling through interaction with Hsp90 (Figs. 3*C–J*), suggesting evolutionary variation within the PIWI subfamily.

Hsp90, 90-kDa heat-shock protein, is a molecular chaperone that contributes to protein folding and stability [50]–[53]. Previous works have indicated that Hsp90 is involved in a wide variety of signaling pathways [36]–[41]. Hsp90 can form complexes with special proteins critical to signal transduction, such as transcription factors and kinases [37], [54]–[56]. Disruption of these complexes leads to degradation of these special client proteins in a proteasome-dependent manner. This affects the signaling pathways that involve Hsp90 [57]–[59]. In the TGF-β signaling pathway, Hsp90 chaperones and stabilizes TβRs, preventing ubiquitin-controlled degradation and ensuring the activation of TGF-β signaling at the receptor level [42].

Recent studies have shown the diversity of HILI functions. HILI can mediate DNA repair and piRNA expression [23], [60], as well as can regulate apoptosis by different pathways including Src-STAT3/p53 and Stat3/Bcl-XL pathway [4], [61]. Cell apoptosis is involved in complicate mechanisms and related to cross-talking of multiple pathways [62]. Our present data showed that a novel regulating pathway of HILI abrogating TGF-β signaling. HILI suppressed TGF-β signaling (Figs. 1*A and 1B*) and also inhibited TGF-β-induced apoptosis (Figs.1*C and 1D*). These data indicate that HILI inhibits apoptosis by suppressing TGF-β signaling.

Here, we present several lines of evidence demonstrating that HILI blocks the formation of the Hsp90-TβR complexes and so weakens TβR stability. This provides some insight into how TGF-β signaling is terminated. First, HILI promotes degradation of TβRs rather than of Smad and blocks TGF-β-induced Smad2/3 phosphorylation and transcription activation (Figs. 1 and 2). Second, ectopic expression of HILI impairs the interaction of Hsp90 with TβRII and TβRI (Figs. 3*A and 3B*). Third, HILI physically associates with Hsp90 *in vivo* and *in vitro*, and is localized in the cytoplasm with Hsp90 (Figs. 3*C–J*). The effects of HILI on TβRs can be rescued by overexpression of Hsp90 (Figs. 3*K and 3L*). Based on evidence given above, we confirm that HILI disrupts the formation of the Hsp90-TβR complexes and promotes TβR degradation by interacting with Hsp90 to inhibit TGF-β signaling. Considering that degradation of TβRII and TβRI are both regulated by ubiquitin E3 ligase Smurf2, we verify that Smurf2 is required for HILI-mediated ubiquitination and degradation of TβRs (Fig. 4) [42], [43].

**Figure 5:**
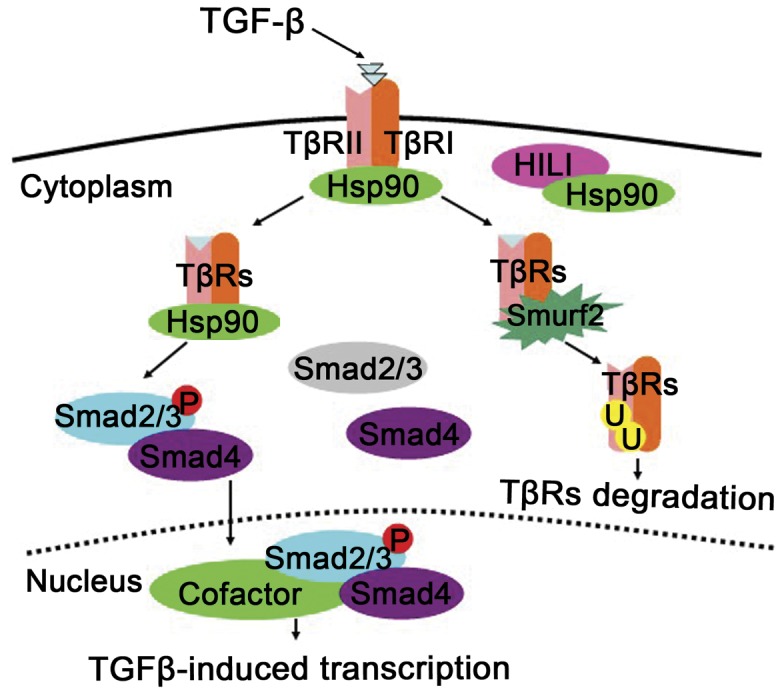
Inhibitive effects of HILI on the TGF-β signaling. HILI competes with TβRs for Hsp90 by binding molecular chaperones, preventing the formation of Hsp90-TβR complexes, and promoting the ubiquitination and degradation of TβRs dependent on the ubiquitin E3 ligase Smurf2. This terminates TGF-β signaling.

In summary, HILI is a novel negative regulator of TGF-β signaling. Our research shows that HILI competes with TβRs for Hsp90 by interacting with this molecular chaperones, prevents formation of Hsp90-TβR complexes, weakens TβR stability, promotes ubiquitination and degradation of TβR in a manner dependent on the ubiquitin E3 ligase Smurf2, and finally blocks Smad2/3 phosphorylation to inhibit TGF-β signaling (Fig. 5). This reveals a critical system of negative regulation of TGF-β signaling. This system is mediated by HILI and its ability to interact with Hsp90. Considering that Hsp90 controls a wide variety of other signaling pathways, this work provides new perspective on the study of the participation of PIWI proteins in regulating diverse types of signal transduction, extending the function of the PIWI subfamily.

## Materials and Methods

### Expression Vectors, HILI Mutants, siRNA, and Antibodies

cDNAs encoding HILI, Hsp90, and ubiquitin, were cloned into pcDNA3.1+Myc or pcDNA3.1+HA expression vector. We constructed various HILI mutants by segmented-PCR and fusion PCR, taking pcDNA3.1Myc-HILI as the template. HD1 was obtained by PCR (Forward primer, F510 5′CGGAATTCCATGGATCCTTTCCGACCATCGTTCA; Reverse primer, R1667 5′GCTCTAGAACTACCGAATGACCTTATGGGAGACATCA). HD2 was obtained by PCR (Forward primer, F510 5′CGGAATTCCATGGATCCTTTCCGACCATCG; Reverse primer, R2042 5′GCTCTAGAACTAGAAGTCCTTCTTCATCTTCTCTGGG). HD3 was obtained by PCR (Forward primer, F1668 5′CGGAATTCCATGAATGACTGTGTGCTGGATGTCATGC; Reverse primer, R3437 5′GCTCTAGAAGTGCAGTCACAGGAAGAACAGGTTC). HD4 (HD4A and HD4B) was obtained by segmented-PCR and fusion PCR. HD4A including N- terminus was first obtained by PCR (Forward primer, F510 5′CGGAATTCCATGGATCCTTTC.

CGACCATCGTTCA; Reverse primer, R1693, 5′CTGAAGTCCTTCTTCATCTTCTCTGGCCG.

AATGACCTTATGGGAGACATC), and HD4B including Piwi domain was obtained by PCR (Forward primer, F2043 5′AGAGCCATGAAGGATTTGGCTCAGC3′; Reverse primer, R3437 5′GCTCTAGAAGTGCAGTCACAGGAAGAACAGGTTC). Finally, HD4 was obtained by fusing HD4A and HD4B by fusion PCR (Forward primer, F510 5′CGGAATTCCATGGATCCT.

TTCCGACCATCGTTCA; Reverse primer, R3437 5′GCTCTAGAAGTGCAGTCACAGGAAG.

AACAGGTTC). HD5 was obtained by PCR (Forward primer, F1534, GGCGAATTCACCATG.

GGCAGAGCCATGAAGGATTTGGCT; Reverse primer, R2868, GGCTCTAGATCAGTGTCC.

TGACAGGAAAGCT). HD6 was obtained by PCR (Forward primer, F1159 GGCGAATTCACC.

ATGGGCAATGACTGTGTGCTGGATGTCAT; Reverse primer, R1512 GGCTCTAGATCATG.

GGATTCCGGTCATAAAA). These mutants were cloned into expression vector pcDNA3.1+Myc. siHILI (sense strand 5′CUA UGA GAU UCC UCA ACU ACA GAAG, antisense strand 5′CUU CUG UAG UUG AGG AAU CUC AUA GUU), siHsp90 (sense strand 5′GGA AAG AGC UGC AUA UUA ATT; antisense stand 5′UUA AUA UGC AGC UCU UUC CTT), siSmurf2 (sense strand 5′GCC CAC ACU UGC UUC AAU CTT, antisense strand 5′GAU UGA AGC AAG UGU GGG CTT), and siControl (non-specific siRNA) were purchased from genepharma. Rabbit anti-Smad2/3 (Santa Cruz Biotechnology), rabbit anti-Smad4 (Santa Cruz Biotechnology), rabbit anti-HILI (Santa Cruz Biotechnology), rabbit anti-HA (Santa Cruz Biotechnology), goat anti-Hsp90 (Santa Cruz Biotechnology), rabbit anti-p21 (Cell Signaling), rabbit anti-phospho-Smad2 (Cell Signaling), rabbit anti-TβRI (Cell Signaling), rabbit anti-TβRII (Cell Signaling), mouse anti-HA (Cell Signaling), rabbit anti-phospho-Smad3 (Epitomics), rabbit anti-Smad7 (HangZhou HuaAn Biotechnology), rabbit anti-PAI-1 (HangZhou HuaAn Biotechnology), rabbit anti-Myc (Abcam), mouse anti-Myc (Zhongshan Goldenbridge), and mouse anti-GAPDH (Boster) were used according to the manufacturers’ instructions.

### Cell Culture, Transfection, and Treatment

HEK-293 cells were maintained in our laboratory as previously described [10]. They were cultured in DMEM/10% FBS and transfected by using Lipofectamine2000 (Invitrogen) according to the manufacturers’ protocols. All transfections were performed in 6-well plates. Forty-eight hours after transfection, cells treatments were carried out in reduced-serum media (0.2% FBS), and TGF-β (Peprotech) was added as indicated to a final concentration of 10 ng/ml. Where specified, cells were treated with the proteasome inhibitor MG132 (Zhongshan Goldenbridge) at a final concentration of 20 µM. Cells were harvested and analyzed by Western blotting using appropriate antibodies. All following experiments were repeated at least three times unless stated otherwise.

### Coimmunoprecipitation and Western Blotting

After HEK-293 cells were transfected with the designated plasmids, cells were lysed in Universal protein extraction buffers (Bioteke). Extracted proteins were immunoprecipitated with special antibody and protein A+G agarose beads (Beyotime). Bound proteins were separated using SDS-PAGE, transferred to polyvinylidene difluoride membranes (Millipore), and detected with specific appropriate primary antibodies and horseradish peroxidase-conjugated secondary antibodies. Specific proteins were visualized using an enhanced chemiluminescence (ECL) Western blot detection system (Amersham Biosciences).

### Apoptosis Assay

Apoptotic rates were analyzed by a COULTER EPICS XL flow cytometer (Beckman, U.S.) by using an Annexin V-EGFP Apoptosis Detection Kit (Bestbio). Annexin V/PI staining and fluorescence intensity measurements were performed according to the manufacturer’s instruction.

### Real-time PCR

Total RNA was prepared using TRIzol (Invitrogen) from HEK-293 cells transfected with Myc-HILI or siHILI. Quantitative PCR was performed in an iCycler IQ real-time PCR Detection System (BioRad, U.S.), with a first denaturation step at 94°C for 10 min, followed by 45 cycles of denaturation at 94°C for 20 s, annealing at 50°C for 30 s, and extension at 72°C for 40 s.

### Immunofluorescence

Transfected cells were fixed for 15 min with 4% formaldehyde in PBS, permeabilized for 10 min with 0.5% Triton X-100, blocked for 30 min with 1% BSA, incubated overnight at 4°C with advisable primary antibody, and finally incubated with FITC-labeled and TRITC-labeled secondary antibody (Zhongshan Goldenbridge) for 1 h at room temperature. Each step was followed by two 5 min washes in PBS. The prepared specimens were counterstained with 5 µg/ml DAPI for 2 min and observed with a fluorescence microscope (Olympus).

### Ubiquitination Assay

HEK-293 cells were transiently cotransfected with vectors encoding HA-ubiquitin (Ub) and Myc-HILI, cultured for 48 h. They were then treated with MG132 for 6 h with TGF-β added for the last 1 h, as indicated. The cells were lysed in universal protein extraction buffers for coimmunoprecipitation and Western blotting. After the lysates were clarified by centrifugation, soluble proteins were coimmunoprecipitated from the cell extracts with anti-TβRI antibody for 2 h and protein A+G agarose beads overnight at 4°C. The beads were washed three times in PBS buffer, and immunoprecipitates were immunoblotted using anti-HA to detect Ub-TβRI.

### In vitro Binding Assays

*In vitro* proteins binding assay was performed using TnT® Quick Coupled *in vitro* transcription/translation system (Promega) according to the manufacturers instructions. *In vitro* cell-free HILI and Hsp90 protein expression were carried out in two reactions using TnT® system, separately. The reactions were carried out in 25 µl volumes by adding 1 µg of plasmid DNA and 1 µl unlabelled methionine to the TnT® mix. Incubate the reaction at 30°C for 90 minutes, and then 2 µl each of the HILI and Hsp90 TnT® reactions were used to detect the protein expressions of HILI and Hsp90 by Western blotting using anti-HILI and anti-Hsp90. Subsequently, 20 µl each of the HILI and Hsp90 TnT® reactions were mixed together in 200 µl binding buffer (20 mM Tris-HCl, pH 7.5, 150 mMNaCl, 1 mM dithiothreitol, 0.1% Tween 20) with protease inhibitors (Complete, EDTA-free, Roche Molecular Biochemicals) and incubated on a rotating platform at 4°C for 3 h. Interaction between HILI and Hsp90 was detected by Co-IP and Western blotting using anti-HILI and anti-Hsp90.

## Supporting Information

Figure S1 **HILI does not bind TβRI.**
(GIF)

Figure S2 **HILI does not bind TβRII.**
(GIF)

Figure S3 **HILI is not a binding partner of Smad2/3/4.**
(GIF)
