# Supplementary figures and images for: HILI Inhibits TGF-β Signaling by Interacting with Hsp90 and Promoting TβR Degradation
Source: PLoS One. 2012 Jul 27;7(7):e41973. doi: 10.1371/journal.pone.0041973 (PMC3407066; doi:10.1371/journal.pone.0041973)

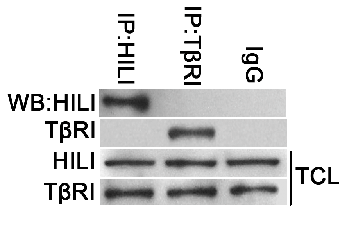

Supplement: Figure S1 — HILI does not bind TβRI. (GIF) [file pone.0041973.s001.gif]

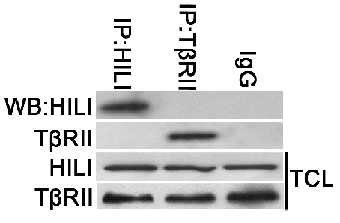

Supplement: Figure S2 — HILI does not bind TβRII. (GIF) [file pone.0041973.s002.gif]

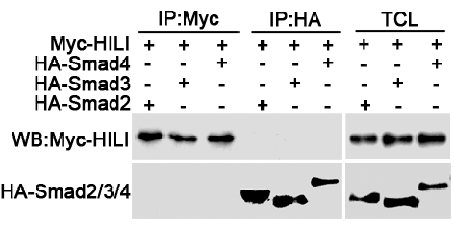

Supplement: Figure S3 — HILI is not a binding partner of Smad2/3/4. (GIF) [file pone.0041973.s003.gif]
